# Supplementary material for: Bistability and time crystals in long-ranged directed percolation
Source: Nat Commun. 2021 Feb 16;12:1061. doi: 10.1038/s41467-021-21259-4 (PMC7886908; doi:10.1038/s41467-021-21259-4)
Supplement: Supplementary file 1 — Supplementary Information [file 41467_2021_21259_MOESM1_ESM.pdf]

Supplementary Information for
"Bistability and time crystals in long-ranged directed percolation"

Andrea Pizzi, Andreas Nunnenkamp, and Johannes Knolle

Supplementary Note 1: Role of the initial density.

First, we investigate our system in yet another direction in the parameter space, that is that of the initial occupation probability (or density) p_1. In the main text, we have in fact seen that the system behaviour in the bistable phase drastically depends on whether p_1 is 'small' or 'large', and, having so far limited our examples to $p_1 = 0.01$ and $p_1 = 1$, we now better assess what do 'small' and 'large' mean. In Supplementary Fig. 1 we consider the entire range of p_1 from 0 to 1, and look for the critical density $p_{1,c}$ separating the two basins of attraction: n does and does not decay to 0 for $p_1 < p_{1,c}$ and $p_1 > p_{1,c}$, respectively. The critical probability at $\alpha \to 0$ coincides with the unstable fixed point x_1 of the equation $x = f_\mu(x)$, as explained in the main text, and is reported in the plots as a reference. For $\mu = 0.8$ (left), we find that $p_{1,c} \approx x_1$ for all the $\alpha \lessapprox 1.5$, whereas for larger α the system enters the inactive phase. For $\mu = 0.9$, we observe that $p_{1,c}$ changes smoothly with α from x_1 at $\alpha = 1$ to 0 at $\alpha \approx 1.6$, when the system enters the active phase, see also Fig. 2(d,e).

As we have show here, the critical probability $p_{1,c}$ of the bistable phase generally falls in the bulk of the range $(0, 1)$, ultimately because the FP x_1 does. This is an important feature of our model, because it means that both the possible behaviours of the bistable phase, that is percolating and not, have a broad range of p_1 in which they are stable. On the one hand, this means that the results of Fig. 1 in the main text are not contingent on the choice of $p_1 = 0.01$ and 1, but would rather be analogue for other choices of $p_1 < p_{1,c}$ and $p_1 > p_{1,c}$. On the other hand, once the Floquet drive is included, such a broad stability region enables the DTC robustness to noise.

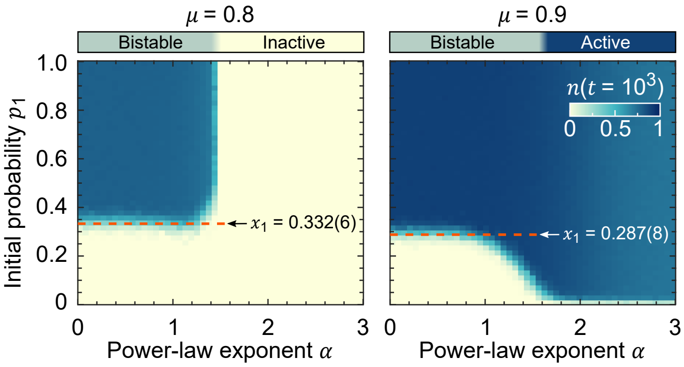

Supplementary Fig. 1. **Role of the initial density in the bistable phase**. Long-time density $n(t = 10^3)$ in the plane of the power-law exponent α and the initial occupation density p_1, for a value of the control parameter $\mu > \mu_c^0 = 0.6550(8)$. For sufficiently small α, the system is in the bistable phase, meaning that $n(10^3)$ can be either finite or not depending on p_1, the two behaviours being separated by a critical probability $p_{1,c}$. For $\alpha \to 0$, the critical probability $p_{1,c}$ corresponds to x_1, the unstable FP of the equation $x = f_\mu(x)$, which is reported as a reference. For $\mu = 0.8$ (left), we observe that $p_{1,c} \approx x_1$ for α up to ≈ 1.5, above which the system enters the inactive phase. For $\mu = 0.9$ (right), instead, the critical $p_{1,c}$ decreases smoothly with α, reaching 0 at $\alpha \approx 1.6$, at which the system enters the active phase. Here, $L = 500$ and $R = 100$.

Supplementary Note 2: Short-range DK limit.

Second, we investigate in more detail the limit $\alpha \to \infty$ already treated in the main text. In such a limit, our model of DP recasts into the DK model, upon replacing $q_1 = f_\mu(0.5) = \mu \tanh(1)$ and $q_2 = f_\mu(1) = \mu \tanh(4)$. Varying μ, one can therefore move across the DK parameter space (q_1, q_2) along the line $q_2 = \frac{\tanh 4}{\tanh 1} q_1$, and therefore across the phase boundary between the active and inactive phases. In Supplementary Fig. 2, we plot the time series of the density n for various values of the control parameter μ. Reiterating from the main text, what we find is that for $\mu = \mu_c^\infty = 0.85(7)$ the density grows as a power law $\sim t^\theta$ with $\theta = 0.3(0)$ as expected in the DP universality class. For $\mu > \mu_c^\infty$ the density n grows in time, whereas for $\mu < \mu_c^\infty$ it rather decays to 0, signalling the active and inactive phases, respectively.

Note that the setting in which DP is studied is usually that of an initial condition with one 'seed site' being occupied, and an infinitely large system size L, corresponding to a density $1/L \to 0$. In our case, the choice of the initial condition with occupation probabilities p_1 corresponds to a possibly small but still finite initial density p_1, so that in the limit $L \to \infty$ there will always be infinitely many seed sites. In the active phase, the clusters originating from many of these sites will grow and expand in time, eventually merging together and leading to a saturation of n at long-times. In particular, the universal power-law growth at criticality can be observed only for $p_1 \ll 1$, and it extends for a finite time, before saturation eventually sets in.

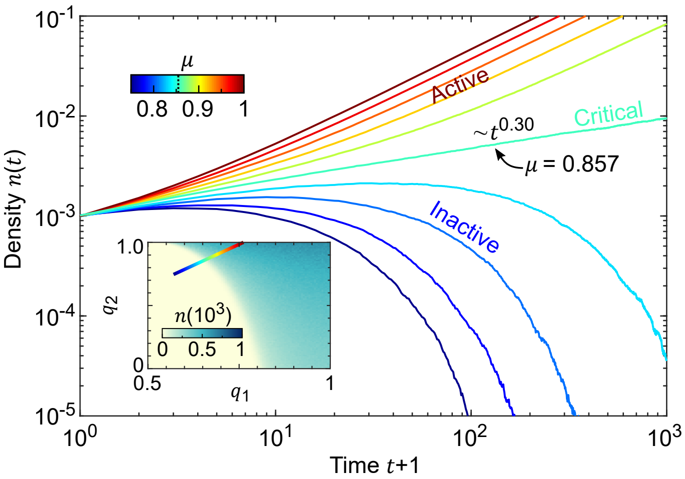

Supplementary Fig. 2. **Limit of short-range directed percolation**. In the short-range limit $\alpha \to \infty$, our model of DP maps to the DK model. In this limit, we show the dynamics of the density n for various values of the control parameter μ. For $\mu < \mu_c^\infty$ ($\mu > \mu_c^\infty$), the system does not (does) percolate, that is n does (does not) decay to 0 at long-times. At the critical point $\mu = \mu_c^\infty \approx 0.857$ (indicated with a dotted line in the colorbar), n grows as a power-law $\sim t^\theta$, with $\theta \approx 0.31$ as expected in the DP universality class. Analogue results are obtained for other choices of the initial density p_1, since in the short-ranged DK limit no bistable phase exists. Here, we consider the following parameters $L = 10^3$, $p_1 = 10^{-3}$ and $R = 10^4$. In the inset, we report for reference Fig. 1(d), highlighting the line spanned in the (q_1, q_2) DK parameter space when varying μ.
